# Serotype-specific role of antigen I/II in the initial steps of the pathogenesis of the infection caused by *Streptococcus suis*

**DOI:** 10.1186/s13567-017-0443-4

**Published:** 2017-07-14

**Authors:** Sarah Chuzeville, Jean-Philippe Auger, Audrey Dumesnil, David Roy, Sonia Lacouture, Nahuel Fittipaldi, Daniel Grenier, Marcelo Gottschalk

**Affiliations:** 1 Swine and Poultry Infectious Diseases Research Center (CRIPA), Saint-Hyacinthe, QC Canada; 2 Groupe de recherche sur les maladies infectieuses en production animale (GREMIP), Department of Pathology and Microbiology, Faculty of Veterinary Medicine, University of Montreal, 3200 Sicotte St., Saint-Hyacinthe, QC J2S 2M2 Canada; 3 Public Health Ontario Laboratory Toronto and Department of Laboratory Medicine and Pathobiology, University of Toronto, Toronto, ON Canada; 4 Oral Ecology Research Group, Faculty of Dentistry, Laval University, Quebec City, QC Canada

## Abstract

*Streptococcus suis* is one of the most important post-weaning porcine bacterial pathogens worldwide. The serotypes 2 and 9 are often considered the most virulent and prevalent serotypes involved in swine infections, especially in Europe. However, knowledge of the bacterial factors involved in the first steps of the pathogenesis of the infection remains scarce. In several pathogenic streptococci, expression of multimodal adhesion proteins known as antigen I/II (AgI/II) have been linked with persistence in the upper respiratory tract and the oral cavity, as well as with bacterial dissemination. Herein, we report expression of these immunostimulatory factors by *S. suis* serotype 2 and 9 strains and that AgI/II-encoding genes are carried by integrative and conjugative elements. Using mutagenesis and different in vitro assays, we demonstrate that the contribution of AgI/II to the virulence of the serotype 2 strain used herein appears to be modest. In contrast, data demonstrate that the serotype 9 AgI/II participates in self-aggregation, induces salivary glycoprotein 340-related aggregation, contributes to biofilm formation and increased strain resistance to low pH, as well as in bacterial adhesion to extracellular matrix proteins and epithelial cells. Moreover, the use of a porcine infection model revealed that AgI/II contributes to colonization of the upper respiratory tract of pigs. Taken together, these findings suggest that surface exposed AgI/II likely play a key role in the first steps of the pathogenesis of the *S. suis* serotype 9 infection.

**Electronic supplementary material:**

The online version of this article (doi:10.1186/s13567-017-0443-4) contains supplementary material, which is available to authorized users.

## Introduction

*Streptococcus suis* is one of the most important post-weaning bacterial pathogens of pigs and a major economic problem for the porcine industry [1]. Septicemia with sudden death, meningitis, arthritis, and endocarditis are the most frequent clinical signs caused by *S. suis* in pigs [2]. *S. suis* is also a zoonotic agent responsible for numerous human cases of meningitis, septicemia, and streptococcal toxic shock-like syndrome [2]. In Western countries, human *S. suis* infections mostly occur in individuals directly or indirectly linked with the porcine industry. In contrast, the general population is at risk of *S. suis* disease in certain Asian countries where this pathogen has been shown to be an important cause of adult meningitis [3]. Serotype 2 is, globally, considered the most virulent serotype and the one most frequently isolated from both porcine and human infections [4]. The use of multilocus sequence typing has revealed that serotype 2 strains belonging to certain sequence types (STs) are more virulent than others. ST1 strains (virulent) predominate in most Eurasian countries, whereas ST25 and ST28 strains (intermediate and low virulence, respectively) are mainly present in North America [4]. Meanwhile, highly virulent ST7 strains, responsible for at least two important human outbreaks in China, have only been reported in that country [5]. The serotype 9 has recently emerged in certain European countries, such as Spain, the Netherlands, and Germany [4]. Yet, very few studies have addressed the presence of virulence factors in this serotype, and putative virulence factors described for serotype 2 strains may not always be present in serotype 9 strains [6]. Moreover, the first *S. suis* serotype 9 human case of infection was reported in 2015 [7].

The early steps of the pathogenesis of the *S. suis* infection are not well understood [1, 8]. Currently, the most accepted hypothesis is that virulent strains reach the bloodstream after breaching the mucosal epithelium of either the upper respiratory or the gastrointestinal tracts of pigs [1]. Similarly, infection of humans occurs via skin wounds or at the intestinal interface following ingestion of raw or undercooked infected meat [1]. However, the precise mechanisms and virulence factors involved remain unknown. Of note, the upper respiratory tract of pigs, particularly the tonsils and nasal cavities, are important reservoirs of *S. suis* [1]. Furthermore, *S. suis* has also been shown to be present in nearly half of the submaxillary lymph node samples of clinically healthy pigs [9]. Bacterial loads in saliva swab and tonsillar brush samples are similar, indicating that *S. suis* is indeed a natural inhabitant of the oral cavity [10].

Antigens I/II (AgI/II) have been extensively described in oral as well as in invasive pathogenic streptococci, including *Streptococcus mutans*, *Streptococcus gordonii*, *Streptococcus pyogenes*, and *Streptococcus agalactiae* [11]. AgI/II are immunostimulatory components and multimodal adhesion proteins implicated in host upper respiratory tract and oral cavity persistence and dissemination [11]. Affinity of AgI/II-like proteins for binding salivary glycoproteins, especially the glycoprotein (gp) 340 (also called DMBT1 protein) is a common feature of this protein family [12]. Large quantities of gp340 are present in the saliva of mammals in either a surface-immobilized form or fluid phase form. It is also present at all mucosal surfaces, including the nasal and intestinal cavities [13, 14]. Interestingly, it has been shown that *S. suis* is able to adhere to gp340 and that this protein aggregates certain strains of *S. suis* [15]. However, the strains tested did not express AgI/II when using a heterologous monospecific antibody [15].

In this study, using in silico analyses, genes with homology to those coding for AgI/II were identified in *S. suis* serotype 2 and 9 strains. Using isogenic mutants deficient for the expression and production of AgI/II in both serotype 2 (S2Δ*agI/II*) and serotype 9 (S9Δ*agI/II*), the role of this protein in different aspects of the pathogenesis of the infection caused by *S. suis* was evaluated. We report for the first time that these proteins play a limited or important role in the pathogenesis of the infection caused by *S. suis* serotype 2 and 9, respectively.

## Materials and methods

### Bacterial strains and culture conditions

Bacterial strains and plasmids used in this study are listed in Table 1. The virulent serotype 2 ST7 strain SC84, responsible for the 2005 human outbreak in China [5], and the serotype 9 strain 1135776 (isolated from a diseased pig in Canada) were used herein as models to study the role of Ag I/II in the pathogenesis of the infection caused by *S. suis*. Twenty-five additional *S. suis* serotype 9 strains recovered from diseased pigs were also used to evaluate the prevalence of *agI/II* genes by PCR (Additional file 1). Seventeen of these strains originated from Canada, 3 from Brazil, 1 from Denmark (reference strain), and 4 from Germany. A strain isolated from a human case of infection was also included [7]. The *S*. *mutans* strain Ingbritt was used as a tool for collection of porcine salivary agglutinins (pSAGs) whereas the *Escherichia coli* TOP10 (Invitrogen, Carlsbad, CA, USA), MC1061 [16], and BL21(DE3) (Invitrogen) strains were used for DNA manipulations and/or AgI/II protein production. The different *Streptococcus* and *E. coli* strains were grown at 37 °C in Todd Hewitt (THB) under static conditions or in Luria-Bertani broth (Becton Dickinson, Franklin Lakes, NJ, USA) with shaking, respectively. Antibiotics (Sigma-Aldrich, St-Louis, MO, USA), where needed, were used at the following concentrations for *S. suis* and *E. coli*: spectinomycin at 500 and 50 µg/mL and erythromycin at 5 and 200 µg/mL, respectively. Ampicillin was also used at a concentration of 50 µg/mL for *E. coli*.

**Table 1:** **Strains and plasmids used in the study**

Strain or plasmid	Characteristics	References
*Streptococcus suis*		
SC84	Serotype 2 strain isolated from a patient with streptococcal toxic shock-like syndrome in China	[47]
1135776	Serotype 9 strain isolated from pig following sudden death in Canada	This study
S2Δ*agI/II*	SC84-derived strain carrying an in-frame deletion of the *agI/II* gene	This study
S9Δ*agI/II*	1135776-derived strain carrying an in-frame deletion of the *agI/II* gene	This study
S2CΔ*agI/II*	SC84-derived strain carrying pOri23-S2*agI/II*	This study
S9CΔ*agI/II*	1135776-derived strain carrying pOri23-S9*agI/II*	This study
*Escherichia coli*		
TOP10	Host for pCR2.1 and pSET4s derivatives	Invitrogen
MC1061	Host for pOri23 derivatives	[16]
BL21(DE3)	Host for pET151 derivatives	Invitrogen
Plasmids		
pET151	Ap^r^, pBR322 *ori*, T7 promotor	Invitrogen
pCR2.1	Ap^r^, Km^r^, pUC *ori*, *lac*ZΔM15	Invitrogen
pSET4s	Spc^r^, pUC *ori*, thermosensitive pG+host3 *ori*, *lac*ZΔM15	[37]
pOri23	Erm^r^, ColE1 *ori*, *P23*	[28]
pET151–S2*agI/II*	pET151 carrying the S2 *agI/II* gene	This study
pSET4s–S2*agI/II*	pSET4s carrying regions upstream and downstream of the S2 *agI/II* gene	This study
pSET4s–S9*agI/II*	pSET4s carrying regions upstream and downstream of the S9 *agI/II* gene	This study
pOri23_spc_–S2*agI/II*	pOri23 carrying the S2 *agI/II* gene as well as its promotor and terminator	This study
pOri23_spc_–S9*agI/II*	pOri23 carrying the S9 *agI/II* gene as well as its promotor and terminator	This study

### Bioinformatics analyses

In silico analyses of AgI/II-coding DNA sequences (CDS) in *S. suis* genomes were performed using BLASTN (expected threshold < 10^−3^) as previously described [17]. The *S.* *suis* nucleotide collection nr/nt database available in GenBank (taxid 1307) was queried for *S. suis* genomes. Alongside, a bank of *S. suis* serotype 2 North American ST25 and ST28 strains isolated from diseased pigs whose genomes were previously published [18, 19] were also queried. Moreover, BLASTN was used to detect homologies with genes coding for AgI/II or orthologues that have already been described in other bacterial species: *S. mutans* SpaP (accession number NC_004350.2), *S. gordonii* SspA and SspB (accession number CP000725.1), *S. pyogenes* (accession number NC_007296.1), *S. agalactiae* (accession number AAJP01000002.1), and *Enterococcus faecalis* (accession number AY855841.2). Examination of CDS carriage by putative integrative and conjugative elements (ICEs) was conducted using the ICEberg database [20], followed by BLASTN using the *S. suis* serotype 2 SC84 (accession number GCA_000026725.1) and serotype 9 D12 (accession number GCA_000231905.1) genomes as queries. Protein domains were analyzed using the NCBI conserved domain database with the help of the BatchCD tool [21]. Cell wall anchored domains were predicted using CW-PRED [22], while transmembrane domains and signal peptide cleavage sites were detected using the TMHMM [23] and the SignalP [24] tools, respectively. The Expasy bioinformatics resource portal was used to determine the theoretical protein molecular weight [25].

### DNA manipulations

Chromosomal *S. suis* DNA was prepared using standard methods [26] or InstaGene matrix (Bio-Rad, Hercules, CA, USA) according to the manufacturer’s instructions. Plasmid DNA preparations and purification of PCR amplicons were performed using the QIAprep Spin Miniprep Kit and the QIAquick PCR Purification Kit (Qiagen, Hilden, Germany), respectively, according to the manufacturer’s instructions. Oligonucleotide primers (listed in Additional file 2) were purchased from Integrated DNA Technologies (Coralville, IA, USA). Primers were designed from the available *S. suis* serotype 2 (strain SC84) and serotype 9 (strain D12) genomes. DNA ligations and transformation of competent *E. coli* were performed as previously described [27]. Sequencing reactions were carried out using an ABI 3730xl Automated DNA Sequencer and the ABI PRISM Dye Terminator Cycle Version 3.1 (Applied Biosystems, Foster City, CA, USA) and analyses of sequences performed using the BioEdit© software and/or BLASTN.

### Generation of the isogenic *agI/II*-deficient mutants and complemented strains

For precise in-frame deletions of the *agI/II* genes in the *S. suis* serotype 2 strain SC84 and serotype 9 strain 1135776, regions upstream and downstream of the genes were amplified and fused by overlap-extension PCR. The amplification products were subcloned into vector pCR2.1 (Invitrogen), excised using *Hin*dIII (Promega, Madison, WI, USA), and cloned into the thermosensitive gene replacement vector pSET4s as previously described [27]. The resulting serotype 2 and serotype 9 pSET4S-*agI/II* vectors were introduced into recipient serotype 2 and 9 strains, respectively. Allelic replacement and absence of AgI/II expression in resulting serotype 2 and serotype 9 *agI/II*-deficient mutants were confirmed by sequencing and Western blot, respectively.

The pOri23 plasmid [28], which carries a gene conferring resistance to erythromycin, was used for complementation assays. A DNA fragment composed of the full sequence of the *agI/II* genes, as well as their putative endogenous promotors and terminators was cloned into pOri23 using the *Eco*RI and *Pst*I restriction enzymes (two constructs, one for the serotype 2 *agI/II* and another for the serotype 9 *agI/II*). Since the serotype 9 strain used is highly resistant to erythromycin (data not shown), and several reports have described increased resistance to this antimicrobial among serotype 2 strains [29, 30], a spectinomycin resistance cassette derived from pSET4s was introduced into the pOri23–S2*agI/II* and pOri23–S9*agI/II* plasmids. Following subcloning steps using *E. coli* MC1061, the generated pOri23_spc_–S2*agI/II* and pOri23_spc_–S9*agI/II* plasmids were then introduced into the S2Δ*agI/II* and S9Δ*agI/II* strains to generate the complemented S2CΔ*agI/II* and S9CΔ*agI/II* strains, respectively.

### Cloning, expression, and purification of the His-tagged recombinant AgI/II protein and production of polyclonal mono-specific antibodies

**Figure 1:**
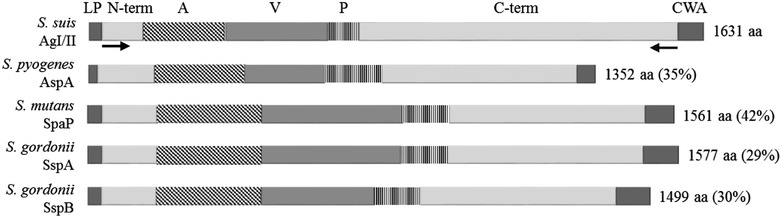
**Characteristics of AgI/II proteins present in different streptococci.** The leader peptide signal (LP), N-terminal domain (N-term), alanine-rich region (A), variable region (V), proline-rich region (P), and C-terminal domain, and the cell wall anchorage domain (CWA) containing the LPXTG domain are illustrated for the *S. suis* AgI/II, *S. pyogenes* AspA, *S. mutans* SpaP, and *S. gordonii* SspA and SspB. Amino acid (aa) size and percentage of *S. suis* AgI/II protein identity are also indicated. Black arrows indicate the location of primers pET151_S2*agI/II*Δ*CWA_*F and pET151_S2*agI/II*_*_*_Δ*LPXTG_*R, which were used to produce the his-tagged recombinant AgI/II protein, rAgI/II.

A 4430 bp fragment of the serotype 2 *agI/II* gene, excluding the sequences coding for the cell wall anchorage and the LPXTG domains, was cloned into the pET151 expression vector (Invitrogen) according to the manufacturer’s instructions (Figure 1). Protein synthesis was induced using 0.5 mM of isopropyl β-d-1-thiogalactopyranoside and cells lysed using lysozyme (Sigma-Aldrich) and sonication. The resulting recombinant His-tagged AgI/II, henceforth rAgI/II, was purified by affinity chromatography using the His-Bind Resin Chromatography Kit (Novagen, Madison, WI, USA,) according to manufacturer’s instructions. Protein purity was evaluated by sodium dodecyl sulfate–polyacrylamide gel electrophoresis following dialysis. Protein concentration was determined using the Pierce Bicinchoninic Acid (BCA) Protein Assay Kit (Thermo Scientific, Waltham, MA, USA). Rabbits were inoculated with the purified rAgI/II to produce a mono-specific polyclonal serum as previously described [31]. This serum was then used to verify presence of the protein in wild-type, isogenic *agI/II*-deficient mutants, and complemented strains by Western blot as previously described [32].

### Cell surface hydrophobicity

The relative surface hydrophobicity of the *S. suis* wild-type strains and *agI/II*-deficient mutants was determined by measuring their adsorption to *n*-hexadecane as previously described [33]. A serotype 2 non-encapsulated mutant strain showing a high percentage of hydrophobicity was used as a positive control [33].

### In vitro pathogenesis assays

#### Self-aggregation and biofilm assays

For the self-aggregation assays, overnight cultures of *S. suis* were washed twice with phosphate-buffered saline (PBS), pH 7.3, and re-suspended in THB to obtain an optical density (OD) at 600 nm of 0.05. Samples were incubated at 37 °C for 24 h under static conditions and self-aggregation quantified as previously described [34]. Biofilm formation capacity was determined as previously described [35] in the absence or presence of 2 mg/mL of porcine fibrinogen (Sigma-Aldrich).

#### *S. suis* aggregation to soluble porcine salivary agglutinins

Saliva was obtained from pigs as previously described [36] with a few modifications. Briefly, cotton ropes were suspended for 30 min to allow a total of 80 growing pigs from a high health status herd with no recent history of endemic *S. suis* disease to chew. No clinical signs of disease were present during collection. Whole saliva was decanted and impurities eliminated by centrifugation at 8000×*g* for 20 min at 4 °C. pSAGs were then purified from clarified saliva as previously described for human salivary agglutinins using *S.* *mutans* [37]. The pSAGs were dialyzed in PBS and the concentration determined using the Pierce BCA Protein Assay Kit. Bacterial aggregation was quantified every 20 min for 1 h in the absence or presence of pSAGs [37].

#### Evaluation of *S. suis* adhesion to extracellular matrix proteins, porcine salivary agglutinins, and the gp340-derived SRCRP2 peptide by ELISA

Bacterial cultures were produced as previously described [38]. Formaldehyde-killed bacteria were washed using either PBS-T (PBS containing 0.05% Tween-20) for experiments involving extracellular matrix proteins (ECM), or TBS-T (10 mM Tris–HCl, 150 mM NaCl, pH 7.5 containing 0.1% Tween-20) supplemented with 1 mM CaCl_2_, for experiments involving pSAGs and the gp340-derived SRCRP2 peptide [39]. Maxisorp flat-bottom microtiter plates (NUNC, Rochester, NY, USA) were coated with 12.5 µg/mL of human plasma fibronectin (Sigma-Aldrich), 15 µg/mL of human type I collagen (Corning, Corning, NY, USA), 1 mg/mL of porcine fibrinogen or 50 µg/mL of pSAGs, all diluted in carbonate coating buffer (0.1 M, pH 9.6), or with 200 µg/mL of the SRCRP2 peptide (Bio Basic Canada Inc., Markham, ON, Canada) diluted in water, overnight at 4 °C. After washing with PBS-T or TBS-T and blocking with non-fat dry milk, bacterial suspensions equivalent to 1 × 10^8^ CFU/mL were added to the plates and incubated at 37 °C for 2 h. Subsequent steps were undertaken as previously described [38] using serotype 2 or 9 specific rabbit antisera and the OD at 450 nm determined.

#### Acid stress killing assay

The ability of *S. suis* to withstand acid challenge was determined as previously described with some modifications [39]. Briefly, *S. suis* strains were grown in THB, washed twice with PBS, and adjusted to a concentration of 1 × 10^8^ CFU/mL. Cells were then resuspended in 0.1 M glycine buffer adjusted to either pH 3.0 or 5.0 and incubated at 37 °C. Surviving bacteria were accurately determined using an Autoplate 4000 Spiral Plater (Spiral Biotech, Norwood, MA, USA).

#### Cell adhesion and invasion assays

The newborn porcine tracheal epithelial cell line (NPTr) was cultured until confluent as previously described [40]. Cells were infected with *S. suis* as previously described with minor modifications [41]. Briefly, PBS-washed NPTr cells were incubated at 37 °C with 5% CO_2_ and infected with *S. suis* at a multiplicity of infection of 10. After 2 h of incubation, wells were washed with PBS to remove non-associated bacteria. For adhesion assays, cells were lysed with 1 mL of cold water, while the invasion assay was performed using the antibiotic protection method as previously described [40], and associated or intracellular bacteria enumerated as described above.

#### Intranasal colonization in a porcine model of infection

All experiments involving animals were conducted in accordance with the guidelines and policies of the Canadian Council on Animal Care and the principles set forth in the Guide for the Care and the Use of Laboratory Animals by the Animal Welfare Committee of the University of Montreal, which approved the protocols and procedures used herein (permit number RECH-1570). Four-week old pigs (providing from the same high health status herd mentioned above) were used. The 10 pigs were randomly separated into two rooms upon arrival and their nasal cavities, saliva, and tonsils swabbed to confirm absence of serotype 9. The *S. suis* serotype 9 wild-type strain 1135776 and *agI/II*-deficient mutant were cultured as previously described [42] to obtain a final concentration of 2 × 10^9^ CFU/mL. Intranasal infections were carried out as previously described with some modifications [43]. Pigs were inoculated with 1 mL of 2% acetic acid per nostril 1 h prior to infection with 1 mL per nostril of either the wild-type or the S9Δ*agI/II* mutant strain.

Nasal cavities were swabbed using sterile cotton-tipped applicators. Swabs were placed in sterile tubes containing PBS supplemented with 0.1% bovine serum albumin and immediately cultured. Serial dilutions of swab samples (10^0^–10^−6^) were plated on Colombia agar supplemented with 5% defibrinated sheep blood (Cedarlane, Burlington, ON, Canada), *Streptococcus* selective reagent SR0126 (Oxoid, Hampshire, UK), and selected antibiotics to which the serotype 9 strain is resistant at the concentrations used (50 μg/mL spectinomycin, 5 μg/mL erythromycin, 0.2 μg/mL penicillin G, and 1 μg/mL tetracycline). After incubation for 24 h at 37 °C with 5% CO_2_, plates containing 30–300 colonies were selected. Suspected alpha-hemolytic colonies were enumerated and 10 *S. suis*-like colonies per plate were sub-cultured and tested by coagglutination assay using anti-*S. suis* serotype 9 rabbit serum as previously described [44]. Three weeks post-infection, pigs were euthanized and tonsils recovered. Tonsil samples were processed as previously described [45] and *S. suis* serotype 9 carriage evaluated as described above.

### Statistical analyses

At least three independent biological replicates were performed for each experiment and results expressed as mean ± standard error of the mean (SEM). Raw data were analyzed using the non-parametric statistical Mann–Whitney test. Statistical differences are defined as being greater than *p* < 0.05.

## Results

### Prevalence and molecular characteristics of the *S. suis* AgI/II

Bioinformatics analyses using the *S. suis* (taxid 1307) genome database available in GenBank revealed the presence of genes coding for AgI/II-like proteins in the genomes of serotype 2 strains, including the ST7 strain SC84, ST1 strain BM407, ST25 strain 89–1591, and in a bank of North American *S. suis* serotype 2 ST25 and ST28 strain genomes [18, 19]. However, they were absent from the genome of the reference ST1 strain P1/7. The gene was also present in the genome of the serotype 9 strain D12. Given the low number of published *S.* *suis* serotype 9 genomes, PCR analyses were undertaken using field strains, which confirmed the presence of the gene in the 25 strains tested (Additional file 1), including strains from Canada, Germany, and Brazil, as well as in the *S. suis* serotype 9 reference strain from Denmark and a human isolate from Thailand. *S. suis* serotype 2 and 9 genes coding for AgI/II share approximately 95% of nucleotide identity. In addition, the promotors share 92% of nucleotide identity with the −35 and −10 boxes and the ribosome binding site for *agI/II* genes being present in all available genomes. Moreover, the terminators of *agI/II* genes are conserved in all strains (100% of nucleotide identity). The percentage of identity between the AgI/II proteins of serotypes 2 and 9 is 95%, being both highly similar. Alignment of the amino acid sequence of both proteins is presented in Additional file 3. Bioinformatics analyses revealed that the *S.* *suis* AgI/II has a theoretical molecular weight of 180 kDa, which is slightly larger than that of other described AgI/II, probably due to the SspB-like isopeptide-forming domain being repeated thrice in the C-terminal part of the *S. suis* AgI/II (Figure 1) [11]. The *S. suis* AgI/II shares between 29 and 42% of protein sequence identity with other streptococcal AgI/II, such as AspA (*S. pyogenes*), SpaP (*S. mutans*), SspA (*S. gordonii*), and SspB (*S. gordonii*) (Figure 1). Alongside, the *S. suis* AgI/II also shares 32% of protein identity with the aggregation substance PrgB (also called Asc10) of *E. faecalis* [46]. The *S. suis* AgI/II has similar characteristic domains to those described in oral streptococci (Figure 1) [11].

Further bioinformatics investigations, including the use of the ICEberg database, revealed that the gene encoding for the AgI/II protein in the serotype 2 strain SC84 is carried by the 89 K ICE (89 Kbp) [47], while that of the serotype 2 ST1 strain BM407 is carried by two putative ICEs annotated as ICE*Ssu*(BM407)1 and ICE*Ssu*(BM407)2) (75 and 80 Kbp, respectively). Moreover, the gene coding for AgI/II in the serotype 9 strain D12 is also carried by an element sharing 95% of nucleotide identity with the whole sequence of ICE*Ssu*(BM407)1. Altogether, these analyses suggest that the *S. suis* AgI/II are mainly carried by ICEs.

### Confirmation of AgI/II-deficient mutants in both *S. suis* serotypes 2 and 9

**Figure 2:**
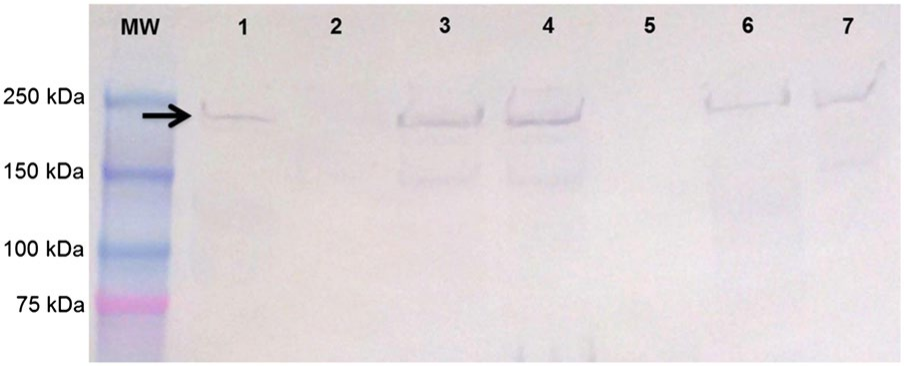
**The AgI/II protein is expressed in the** ***S. suis*** **serotype 2 and 9 wild-type strains but is absent in S2Δ** ***agI/II*** **and S9Δ** ***agI/II*** **mutant strains.** Western blot using cell wall extracts from *S. suis* serotype 2 (wells 1–3) and serotype 9 (wells 4–6): serotype 2 wild-type strain SC84 (well 1) and serotype 9 wild-type strain 1135776 (well 4); mutant strains S2Δ*agI/II* (well 2) and S9Δ*agI/II* (well 5); and complemented strains S2CΔ*agI/II* (well 3) and S9CΔ*agI/II* (well 6). Expected bands at approximately 180 kDa, shown by the black arrow, were observed for the serotype 2 and 9 wild-type and complemented strains, similar to that obtained with the purified AgI/II protein, rAgI/II (well 7), used as a positive control. MW: molecular weight marker.

Production of AgI/II by the serotype 2 and 9 strains SC84 and 1135776, respectively, was confirmed by immunoblotting using mono-specific antisera produced with the recombinant protein, rAgI/II (Figure 2). The proteins had a molecular weight of approximately 180 kDa, as predicted by bioinformatics analyses. Deletion of the *agI/II* gene resulted in absence of detectable signal while complementation of the mutant strains restored detection with a band at the expected molecular weight (Figure 2). Growth of the S2Δ*agI/II* and S9Δ*agI/II* mutants as well as that of the complemented strains was similar to their respective wild-type strains (data not shown).

It was previously described that AgI/II positively impacts surface hydrophobicity of oral streptococci. However, we did not observe significant differences in hydrophobicity between the *S. suis* serotype 2 or 9 wild-type strains and their AgI/II-deficient mutants (S2Δ*agI/II* and S9Δ*agI/II*) (Additional file 4). Interestingly, the serotype 2 wild-type strain was significantly more hydrophobic than that of serotype 9 (*p* < 0.05).

### In vitro pathogenesis assays

#### Serotype-dependent role of the *S. suis* AgI/II in self-aggregation and biofilm formation

**Figure 3:**
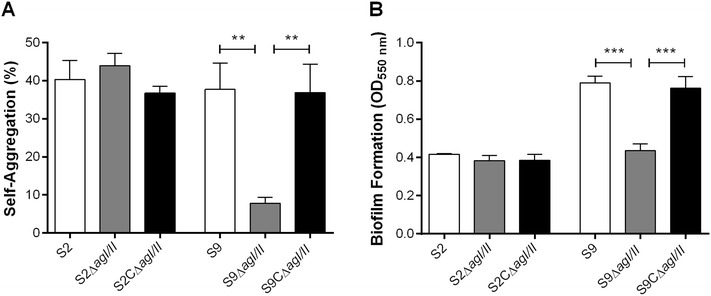
**The** ***S. suis*** **serotype 9 (S9) AgI/II, but not that of the serotype 2 (S2), is implicated in bacterial self-aggregation and biofilm formation.** The role of the *S. suis* AgI/II was evaluated with regards to cell-to-cell aggregation in fluid phase (**A**) and biofilm formation capacity in the presence of porcine fibrinogen (**B**) after 24 h of incubation at 37 °C. Data represent the mean ± SEM from at least three independent experiments. **(*p* < 0.01) and ***(*p* < 0.001) indicate a significant difference between the *S. suis* S9 wild-type or complemented strain (S9CΔ*agI/II*) and *agI/II*-deficient mutant (S9Δ*agI/II*).

*S. suis* serotype 2 self-aggregation was not modified by the absence of AgI/II (Figure 3A). However, deletion of AgI/II significantly reduced self-aggregation of *S. suis* serotype 9 by 80% (*p* < 0.01) (Figure 3A). On the other hand, self-aggregation was completely restored when using the complemented S9CΔ*agI/II* strain (Figure 3A). Thus, the serotype 9 AgI/II, but not that of serotype 2, is involved in bacterial self-aggregation.

The role of AgI/II in biofilm formation was evaluated for both serotype 2 and 9 in the presence of porcine fibrinogen. The capacity of the serotype 2 strain to form biofilm was relatively low, and no difference was observed in the absence of AgI/II (Figure 3B). On the other hand, the serotype 9 wild-type strain showed a significantly greater capacity to form biofilm than the wild-type serotype 2 strain in the presence of porcine fibrinogen (*p* < 0.01). Furthermore, the serotype 9 AgI/II was significantly involved in this bacterial function (*p* < 0.001) (Figure 3B). The capacity to form biofilm was restored in the complemented S9CΔ*agI/II* strain (Figure 3B). Minimal biofilm formation was observed in the absence of porcine fibrinogen for both the serotype 2 and 9 strains (Additional file 5). Consequently, the serotype 9 AgI/II, but not that of serotype 2, plays an important role in the capacity to form biofilm.

#### The *S. suis* AgI/II increases both porcine salivary agglutinin induced-aggregation and adhesion to salivary agglutinins

Salivary agglutinins are major receptors of streptococcal AgI/II [12]. Thus, we investigated the interactions of the *S. suis* serotype 2 and 9 AgI/II with fluid phase (miming the conditions in saliva) and surface-immobilized (miming mucosa such as in the oral cavity) pSAGs. pSAGs collected from pig saliva was obtained at a concentration of 50 μg/mL, which is similar to that usually obtained for human salivary agglutinins [37].

**Figure 4:**
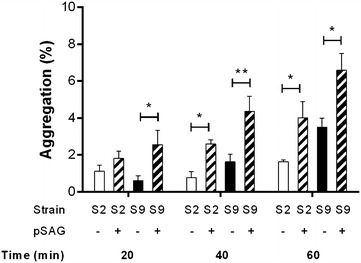
**Porcine salivary agglutinins (pSAGs) aggregate** ***S. suis*** **serotype 2 (S2) and serotype 9 (S9).** Evaluation of the fluid phase aggregation of the wild-type *S. suis* S2 and S9 strains in the absence (−) or presence (+) of pSAGs. Aggregation in the absence of pSAGs reflects self-aggregation only. Data represent the mean ± SEM from at least three independent experiments. *(*p* < 0.05) and **(*p* < 0.01) indicate a significant difference of *S. suis* S2 or S9 aggregation in the absence and presence of pSAGs.

**Figure 5:**
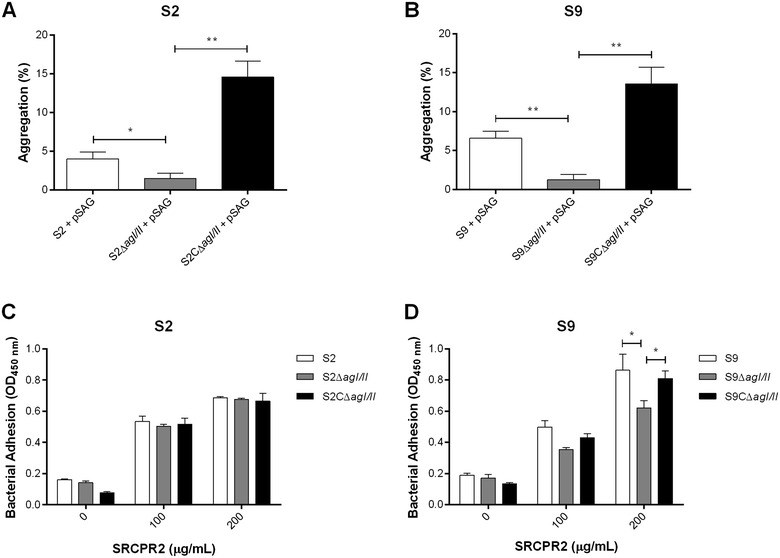
**The** ***S. suis*** **serotype 2 (S2) and serotype 9 (S9) AgI/II are involved in adhesion to fluid phase porcine salivary agglutinins (pSAGs), but only for S9 with surface-immobilized pSAGs.** Evaluation of the fluid phase aggregation of S2 (**A**) and S9 (**B**) strains to pSAGs or to surface-immobilized gp340-derived peptide SRCRP2 by S2 (**C**) and S9 (**D**), the latter being measured by ELISA. Data represent the mean ± SEM from at least three independent experiments. *(*p* < 0.05) and **(*p* < 0.01) indicate a significant difference between the *S. suis* S2 or S9 wild-type or complemented strain (S2CΔ*agI/II* or S9CΔ*agI/II*) and the *agI/II*-deficient mutants (S2Δ*agI/II* or S9Δ*agI/II*).

Results showed a significantly more rapid and greater aggregation of both *S. suis* serotype 2 or serotype 9 strains in the presence of pSAGs (*p* < 0.05) (Figure 4). Moreover, this fluid phase pSAG-induced aggregation significantly increased with time (*p* < 0.05) (Figure 4). However, the pSAG-mediated aggregation induced by the serotype 9 strain was significantly higher than that induced by the serotype 2 strain, but only after 60 min of incubation (*p* < 0.05) (Figure 4). AgI/II-deficiency significantly reduced fluid phase pSAG-induced aggregation for both serotypes (*p* < 0.05) (Figures 5A and B), and complementation of AgI/II-deficient mutants restored fluid phase pSAG-induced aggregation (*p* < 0.01) (Figures 5A and B).

The adhesion of *S. suis* to surface-immobilized pSAGs was then evaluated using ELISA. Since background obtained with crude pSAGs was very elevated (data not shown), the gp340-derived peptide SRCRP2, described as the major binding sequence for AgI/II [37], was used. Results showed that deletion of the *S. suis* serotype 2 *agI/II* had no effect on adhesion to SRCRP2 (Figure 5C), while that of serotype 9 significantly reduced adhesion to SRCRP2 (*p* < 0.05), but only at a concentration of 200 μg/mL (Figure 5D). As expected, complementation of the *S. suis* serotype 9 AgI/II-deficient mutant restored adhesion to SRCRP2 (Figure 5D).

Taken together, these results demonstrate that AgI/II promotes pSAG-induced aggregation when in fluid phase for both serotypes, and adhesion to the gp340-derived peptide SRCRP2 at a high concentration for serotype 9 only.

#### The *S. suis* AgI/II confers protection to acid stress

**Figure 6:**
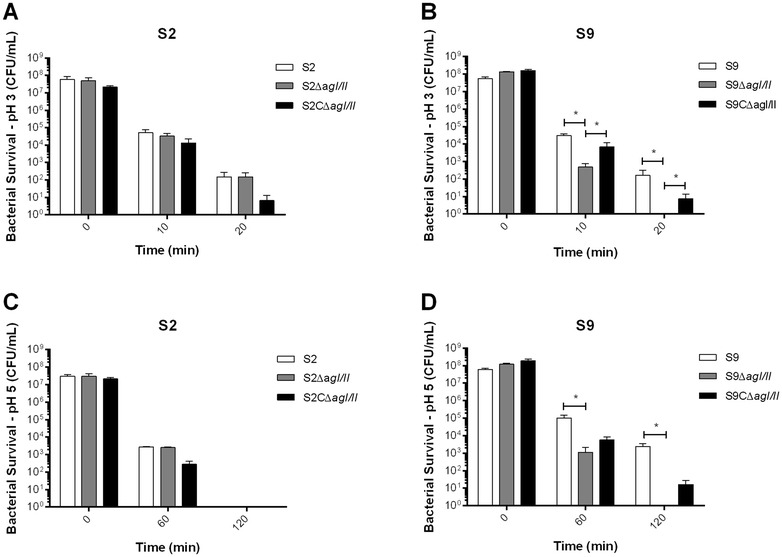
**The** ***S. suis*** **serotype 9 (S9) AgI/II, but not that of serotype 2 (S2), is involved in protection against acid stress.** Effect of acid stress on *S. suis* S2 and S9 viability, determined at pH 3 (**A**, **B**) and pH 5 (**C**, **D**). Data represent the mean ± SEM from at least three independent experiments. *(*p* < 0.05) indicates a significant difference between the *S. suis* S9 wild-type or complemented strain (S9CΔ*agI/II*) and *agI/II*-deficient mutant (S9Δ*agI/II*).

Once swallowed, *S. suis* will reach the stomach, in which it must overcome hostile environmental conditions such as low pH. We thus investigated the role of AgI/II and aggregation in resistance to low pH. Acid stress killing assays revealed that the *S. suis* serotype 2 AgI/II was not involved in acid resistance at pH 3 (Figure 6A) nor at pH 5 (Figure 6C). On the other hand, results showed that the S9Δ*agI/II* mutant strain survived significantly less than its wild-type strain (*p* < 0.05) at both pH 3 (Figure 6B) and pH 5 (Figure 6D). Thus, AgI/II confers partial protection to *S. suis* serotype 9, but not to serotype 2, against acidic environments.

#### The *S. suis* serotype 9 AgI/II contributes to adhesion to extracellular matrix proteins and to porcine epithelial cells

**Figure 7:**
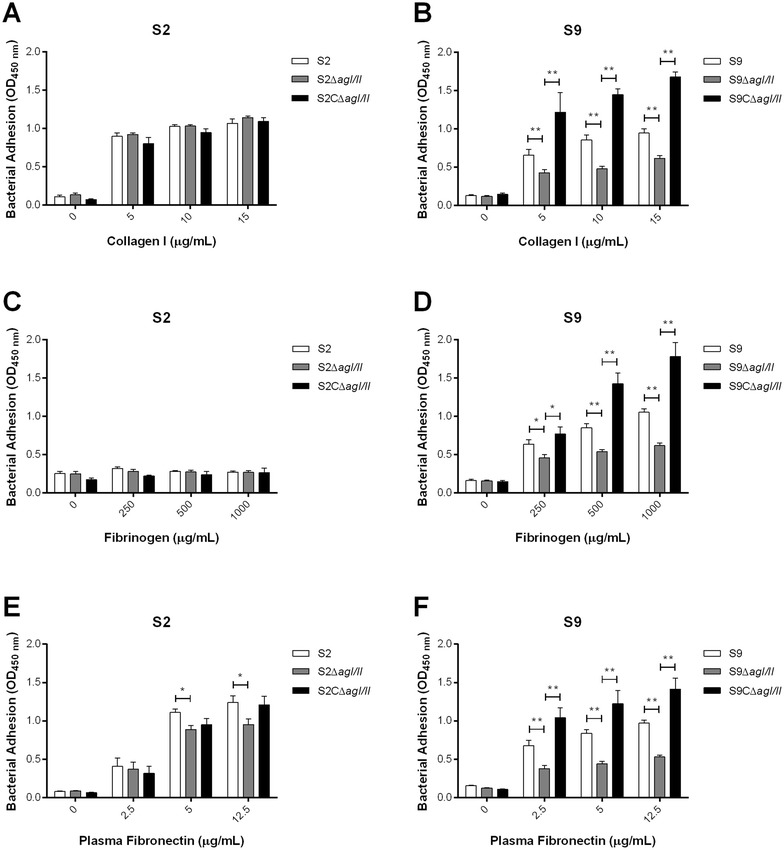
**The** ***S. suis*** **serotype 9 (S9) AgI/II and, to a lesser extent, that of serotype 2 (S2), are bacterial adhesins for extracellular matrix proteins.** Adhesion of the *S. suis* S2 and S9 strains to different concentrations of collagen I (**A**, **B**), fibrinogen (**C**, **D**), and plasma fibronectin (**E**, **F**) as evaluated by ELISA. Data represent the mean ± SEM from at least three independent experiments. *(*p* < 0.05) and **(*p* < 0.01) indicate a significant difference between the wild-type or complemented strain (CΔ*agI/II*) and the *agI/II*-deficient mutant (Δ*agI/II*).

AgI/II was previously described in other streptococci as binding ECM proteins and contributing to adhesion to and invasion of epithelial cells. Our results showed that while the serotype 2 AgI/II was not involved in adhesion to collagen I, that of the serotype 9 played a significant role (*p* < 0.01) (Figures 7A and B). In accordance, complementation of the S9Δ*agI/II* mutant restored the wild-type phenotype (Figure 7B). Moreover, as previously described with other serotype 2 strains [38], the serotype 2 wild-type strain used in this study (SC84) did not bind porcine fibrinogen (Figure 7C). On the other hand, the serotype 9 wild-type strain did bind to porcine fibrinogen, with absence of AgI/II significantly reducing this ability (*p* < 0.05) (Figure 7D). Once again, complementation of the S9Δ*agI/II* mutant strain restored this adhesion capacity (Figure 7D). Finally, the deletion of the *S. suis* serotype 9 *agI/II* gene and, to a lesser extent, that of the serotype 2, significantly decreased adhesion to plasma fibronectin (*p* < 0.05) (Figures 7E and F). Consequently, these results demonstrate the importance of AgI/II as a multimodal adhesin for *S. suis* serotype 9 while only playing a minor role for serotype 2.

**Figure 8:**
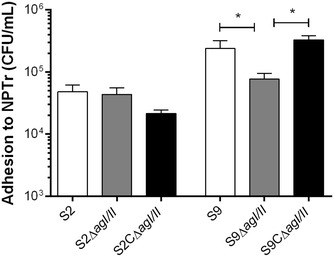
**The** ***S. suis*** **serotype 9 (S9) AgI/II, but not that of the serotype 2 (S2), is involved in adhesion to porcine tracheal epithelial cells.** Adhesion of the *S. suis* S2 and S9 strains to NPTr cells after 2 h of incubation with a multiplicity of infection of 10. Data represent the mean ± SEM from at least three independent experiments. *(*p* < 0.05) indicates a significant difference between the *S. suis* S9 wild-type or complemented strain (S9CΔ*agI/II*) and the *agI/II*-deficient mutant (S9Δ*agI/II*).

The role of AgI/II in adhesion to and invasion of porcine tracheal epithelial cells was subsequently investigated. Interestingly, the serotype 9 wild-type strain adhered significantly more to epithelial cells than did the serotype 2 (*p* < 0.05) (Figure 8). Adhesion assays revealed a significant decrease in adhesion to epithelial cells in the absence of AgI/II for the serotype 9 (*p* < 0.05), equivalent to 30% of wild-type strain adhesion, with complementation restoring adhesion (Figure 8). On the other hand, no differences were observed between the *S. suis* serotype 2 wild-type strain and its AgI/II-deficient mutant (Figure 8). Low levels of epithelial cell invasion were observed for both serotypes, with no role of AgI/II being evident (data not shown). Taken together, these results reveal that AgI/II is implicated in adhesion to host proteins and epithelial cells for serotype 9 and, to a lesser extent, for serotype 2.

#### Role of AgI/II in colonization of the oral and nasal cavities of pigs

**Figure 9:**
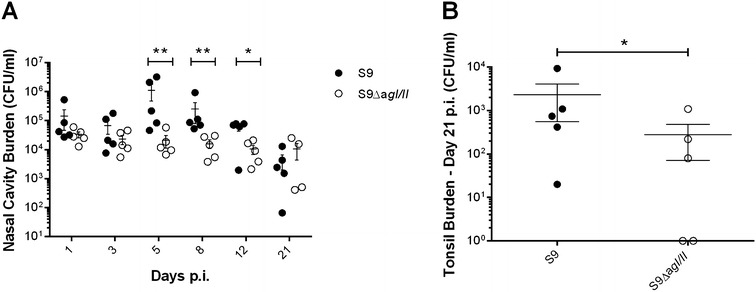
**The** ***S. suis*** **serotype 9 (S9) AgI/II is implicated in colonization of the porcine respiratory tract.** An intranasal porcine model of infection was used to determine the implication of the *S. suis* S9 AgI/II in colonization of the nasal cavity (**A**) and tonsils 21 days post-infection (**B**). Data represent the mean ± SEM from at least three independent experiments. *(*p* < 0.05) and **(*p* < 0.01) indicate a significant difference between presence of the *S. suis* S9 wild-type strain and the *agI/II*-deficient mutant (S9Δ*agI/II*).

Given that in vitro results demonstrated an important role of AgI/II for *S. suis* serotype 9, we next evaluated the contribution of this protein in colonization using a porcine infection model. Animals were divided into two groups and infected with either the serotype 9 wild-type strain or the AgI/II-deficient mutant by intranasal inoculation. Evaluation of serotype 9 colonization revealed that the number of wild-type strain recovered from the nasal cavities significantly increased over time until day 12 post-infection (p.i.) (*p* < 0.05), whereas the number of S9Δ*agI/II* remained stable throughout the experiment (Figure 9A). Moreover, AgI/II-deficient mutants were recovered in significantly lower numbers from the nasal cavities of pigs on days 5, 8, and 12 p.i. (*p* < 0.05) (Figure 9A). Although the number of serotype 9 wild-type strain and AgI/II-deficient mutant in the nasal cavities of pigs was similar 21 days p.i. (Figure 9A), AgI/II-deficiency resulted in significantly reduced colonization of tonsils (*p* < 0.05) (Figure 9B). Together, these results strongly suggest that the serotype 9 AgI/II contributes to colonization of the porcine respiratory tract.

## Discussion

AgI/II proteins have been extensively described in oral pathogenic streptococci as multimodal adhesion proteins and immunostimulatory components implicated in host upper respiratory tract and oral cavity persistence and dissemination [11]. In addition, it has been shown that AgI/II proteins potentially play multiple roles in *Streptococcus* adherence, colonization, and microbial community development [11]. These proteins have also been described in pyogenic streptococci, such as *S. pyogenes* and *S. agalactiae,* but they have never been identified in *Streptococcus pneumoniae* [11]. An initial goal of this study was to determine whether *S. suis* possesses these putative virulence factors. We showed that most of the *S. suis* serotype 2 available genomes, including from different STs, possess genes encoding AgI/II. Interestingly, the gene was absent from the ST1 strain P1/7, which is commonly used as a reference for investigation of virulence [6]. We also identified AgI/II-encoding genes in the genome of the Chinese serotype 9 strain D12, in the serotype 9 reference strain 22083, as well as in a collection of 25 serotype 9 field strains (added herein given the limited number of serotype 9 genomes available), alongside a human isolate, tested by PCR.

It is widely recognized that mobile genetic elements such as insertion sequences, transposons, bacteriophages, plasmids, and genomic islands are key drivers of genomic evolution and bacterial adaptation. Among them, ICEs are chromosomal genetic elements that play an important role in horizontal gene transfer [48]. In both *S. pyogenes* and *S. agalactiae*, AgI/II are encoded by genes carried by ICEs, which can spread not only to other *S. pyogenes* and *S.* *agalactiae* strains, but also to other streptococci [49, 50]. Meanwhile, different ICEs have been described in *S. suis* [51], of which the 89 K ICE carried by the *S.* *suis* serotype 2 strain SC84 has been suggested to be responsible, at least in part, for the higher virulence of this strain [52]. Interestingly, results obtained in this study showed that the *S. suis agI/II* genes are mainly carried by ICEs. As such, it may be suggested that acquisition of AgI/II by *S. suis* occurred via horizontal transfer following acquisition of ICEs.

Persistence of *S. suis* in the oral cavity may contribute to the pathogenesis of the infection. Our data showed that AgI/II plays an important role in self-aggregation for *S. suis* serotype 9. This role was even more important in the presence of salivary glycoproteins, such as gp340. It has been previously shown that human salivary gp340 was able to aggregate an untypeable, a serotype 1, and a serotype 2 *S. suis* strain [15]. However, these strains were negative for the expression of AgI/II as evaluated by immunoblot using a polyclonal antibody raised against the *S. mutans* proteins [15]. In the present study, we showed that purified soluble pSAGs increase the ability of *S. suis* to aggregate and that AgI/II played an important role in such interactions for serotype 9 and, to a lesser extent, serotype 2. Fluid phase and surface-immobilized gp340 expose different binding properties and, consequently, differentially recognize adhesive phenotypes of diverse bacterial species. Herein, we showed that AgI/II also played a role in the *S. suis* serotype 9 adhesion to the surface-immobilized gp340-derived peptide SRCRP2. Similarly, the AgI/II from *S. suis* serotype 9 also played an important role in biofilm formation.

The relationship between the saliva-dependent aggregation, attachment to salivary glycoproteins, and biofilm formation in the oral cavity and pathogenesis of the infection caused by pathogenic streptococci is not very clear. On the one hand, aggregation (clumping) may presumably allow “bacterial clearance” from the oral cavity via swallowing [53]. It is usually accepted that the main route of infection for pigs is through the respiratory tract. However, more recently, the oral route (as clearly described in humans) has also been suggested as a portal of entry in pigs [54]. Although a recent report showed that disease could not be induced in an experimental infection by the oral route in post-weaned animals [55], a role of early colonization of the intestine of pre-weaned piglets followed by direct invasion through intestinal epithelial cells in animals under post-weaned stress could not be completely ruled out [1]. In the present study, an increased susceptibility to low pH (usually found in the stomach) was observed for *S. suis* serotype 9 in the absence of the *agI/II* gene. As such, it may be hypothesized that AgI/II induces bacterial self-mediated and salivary agglutinin-mediated aggregation and biofilm formation for serotype 9, which would increase, at certain moments, the swallowing of large amounts of bacteria. AgI/II would subsequently increase bacterial protection against the low pH of the stomach, thus allowing colonization of the intestine. However, this hypothesis remains to be confirmed.

It has been proposed that adhesion to epithelial cells is one of the most important initial steps of the pathogenesis of the infection caused by *S. suis* [1]. Similarly to other pathogens, *S. suis* is also able to bind ECM components, which have been suggested to be implicated as cell receptors [1]. At least 28 different *S. suis* components have been described to be involved in such interactions so far [1, 6]. In the present study, it was clearly shown that the AgI/II plays an important role in the adhesion of *S. suis* serotype 9 to collagen I, fibrinogen, and fibronectin. In the case of serotype 2, this protein plays a minimal role in adhesion to fibronectin and none to collagen I. As previously described, the serotype 2 strain was unable to bind fibrinogen [38]. The lack of binding to the latter may also explain differences observed in biofilm formation (in the presence of this protein) between serotype 2 and serotype 9 strains and the important role played by the serotype 9 AgI/II.

The implication of AgI/II in the adhesion to epithelial cells was further evaluated using porcine tracheal epithelial cells as a model [40]. Firstly, it was interesting to note that the serotype 9 wild-type strain presented higher adhesion levels than the serotype 2 strain, a fact that has been previously reported with other porcine cells [54]. A role was attributed to AgI/II in the adhesion of serotype 9 since a significant reduction of adhesion to these cells was observed using the S9Δ*agI/II* mutant. This reduction of adhesion could be explained by a reduction in the interactions with ECM components (as described above) or through a direct effect of the AgI/II as an adhesin. In fact, this protein has been described to be directly involved in epithelial cell adhesion and invasion by *S. gordonii* through β1 integrin recognition [56]. Using a different mechanism, this protein was also involved in adhesion/invasion of *S.* *pyogenes* to these cells [56].

Previous studies showed that the *S. pyogenes* AgI/II is implicated in upper respiratory tract colonization [57]. Since results showed that AgI/II plays important roles in vitro for serotype 9, its implication in colonization of the upper respiratory tract was investigated in pigs. As previously described, pigs infected by the serotype 9 wild-type strain and its isogenic S9Δ*agI/II* mutant via the intranasal route did not develop clinical signs of infection [43]. However, a slight, yet significantly lower colonization of the upper respiratory tract by the mutant strain, and, subsequently at the tonsillar level, was observed, suggesting that this protein may collaborate in bacterial colonization during the first steps of the infection. However, additional studies should be carried out to confirm this hypothesis.

In conclusion, the presence of AgI/II is herein reported for the first time in *S. suis*. This protein appears to play important or limited roles during the first steps of the pathogenesis of the infection caused by serotypes 9 and 2, respectively. Since the gene and protein sequences are highly similar between both serotypes, the observed differences are more difficult to explain than anticipated, and several hypotheses may be proposed. Firstly, a particular motif specific to the gene coding for the serotype 9 AgI/II might be responsible for the phenotypic differences highlighted in this study. Secondly, the *S. suis* serotype 2 and 9 *agI/II* genes are both carried by ICEs, which vary, creating differing genetic contexts and, consequently, differential gene regulation. Thirdly, critical *S. suis* virulence factors still remain poorly known [6]; the lack of a dominant role of the serotype 2 AgI/II observed herein might also be due to compensation by other virulence factors that result in bacterial redundancy [6]. Further studies are presently underway to explore these avenues. Overall, AgI/II may contribute to the colonization of the upper respiratory tract of pigs and could represent important surface bacterial components implicated in the first steps of the pathogenesis of the infection caused by *S. suis*.

## Additional files

**Additional file 1.** **List of** ***S. suis*** **serotype 9 strains used in this study and their characteristics.**

**Additional file 2.** **List of primers used in this study.** Restriction sites are underlined and in bold.

**Additional file 3.** ***S. suis*** **serotype 2 (S2) and serotype 9 (S9) AgI/II amino acid sequence alignment.** Alignment was performed using Vector NTI 11.5. Conserved amino acids appear in light gray and identical amino acids in dark gray.

**Additional file 4.** **Percent hydrophobicity of the** ***S. suis*** **serotype 2 (S2) and serotype 9 (S9) wild-type and** ***agI/II*** **-deficient mutant strains.** Hydrophobicity was determined using *n*-hexadecane and the non-encapsulated *S. suis* serotype 2 strain, S2Δ*cpsF*, included as a positive control. Data represent the mean ± SEM from at least three independent experiments.

**Additional file 5.** **Biofilm formation by the** ***S. suis*** **serotype 2 (S2) and serotype 9 (S9) wild-type and** ***agI/II*** **-deficient mutant strains in the absence of porcine fibrinogen.** Biofilm formation capacity was quantified after 24 h of incubation at 37 °C in the absence of porcine fibrinogen. Data represent the mean ± SEM from at least three independent experiments.

## Abbreviations

AgI/II: antigen I/II
BCA: bicinchoninic acid
CDS: coding DNA sequence
ECM: extracellular matrix protein
gp340: glycoprotein 340
ICE: integrative and conjugative element
NPTr: newborn porcine tracheal epithelial cell
OD: optical density
PBS: phosphate-buffered saline
p.i.: post-infection
pSAG: porcine salivary agglutinin
SEM: standard error of the mean
ST: sequence type
THB: Todd Hewitt broth
