# Supplementary material for: Serotype-specific role of antigen I/II in the initial steps of the pathogenesis of the infection caused by Streptococcus suis
Source: Vet Res. 2017 Jul 14;48:39. doi: 10.1186/s13567-017-0443-4 (PMC5513104; doi:10.1186/s13567-017-0443-4)
Supplement: Supplementary file 1 — Additional file 1. List of S. suis serotype 9 strains used in this study and their characteristics. [file 13567_2017_443_MOESM1_ESM.docx]

**Additional File 1. List of *S. suis* serotype 9 strains used in this study and their characteristics.**

| **Strain** | **Country** | **Host Origin** | **Tissue Origin** |
| --- | --- | --- | --- |
| 1388970 | Canada | Pig | Brain |
| 1370475 | Canada | Pig | Heart |
| 1509635 | Canada | Pig | Brain |
| 1439272 | Canada | Pig | Spleen |
| 1406687 | Canada | Pig | Lung |
| 1398038 | Canada | Pig | Heart |
| 1358915 | Canada | Pig | Unknown |
| 1355868 | Canada | Pig | Liver |
| 1275845 | Canada | Pig | Brain |
| 1273590 | Canada | Pig | Lung |
| 1142943 | Canada | Pig | Brain |
| 1135776 | Canada | Pig | Spleen |
| 1136450 | Canada | Pig | Spleen |
| 1137833 | Canada | Pig | Spleen |
| 1130349 | Canada | Pig | Spleen |
| 1129705 | Canada | Pig | Spleen |
| 1092236 | Canada | Pig | Kidney |
| 22083 | Denmark | Pig | Brain |
| 1580443 | Germany | Pig | Blood |
| 1580444 | Germany | Pig | Meninges |
| 1580445 | Germany | Pig | Unknown |
| 1580446 | Germany | Pig | Unknown |
| 1135/10 | Brazil | Pig | Brain |
| 1136/10 | Brazil | Pig | Spleen |
| 1016/10 | Brazil | Pig | Spleen |
| 89-289 | Canada | Pig | Brain |
| 1584695 | Thailand | Human | Blood |
