# Supplementary material for: Serotype-specific role of antigen I/II in the initial steps of the pathogenesis of the infection caused by Streptococcus suis
Source: Vet Res. 2017 Jul 14;48:39. doi: 10.1186/s13567-017-0443-4 (PMC5513104; doi:10.1186/s13567-017-0443-4)
Supplement: Supplementary file 2 — Additional file 2. List of primers used in this study. Restriction sites are underlined and in bold. [file 13567_2017_443_MOESM2_ESM.docx]

**Additional File 2. List of primers used in this study.** Restriction sites are underlined and in bold.

| **Name** | **Sequence** | **Use** |
| --- | --- | --- |
| Scr_S9*agI/II*___F | AGAGACAATTGCACGAGGCT | Screening of *agI/II* gene in serotype 9 strains |
| Scr_S9*agI/II*___R | GTGGGTCCTCAGGCATGAAA | Screening of *agI/II* gene in serotype 9 strains |
| S2Δ*agI/II_*1 | GAGTTGCTGCTTCACAGTA | Construction of S2Δ*agI/II* mutant |
| S2Δ*agI/II_*2 | ACTCTTACGAAAGACGTGTT | Construction of S2Δ*agI/II* and S9Δ*agI/II* mutants |
| S2Δ*agI/II_*3 | TGCCAAATACAGGGGAACA | Construction of S2Δ*agI/II* and S9Δ*agI/II* mutants |
| S2Δ*agI/II_*4 | AGGACTAGCCTGGACAAA | Construction of S2Δ*agI/II* and S9Δ*agI/II* mutants |
| S2Δ*agI/II_*5 | TTTTT**AAGCTT**TATCTTCTGAGAGTGTTATTTGAT | Construction of S2Δ*agI/II* and S9Δ*agI/II* mutants |
| S2Δ*agI/II_*6 | TAGAATACCAGCTCCAATAAAGTGTCCTTCTTTTCTTTTTTC | Construction of S2Δ*agI/II* mutant |
| S2Δ*agI/II_*7 | AGAAAAGAAGGACACTTTATTGGAGCTGGTATTCTACTTGGTA | Construction of S2Δ*agI/II* mutant |
| S2Δ*agI/II_*8 | TTTTT**AAGCTT**CTGAACAGATCTTTTGATTCCCT | Construction of S2Δ*agI/II* mutant |
| S9Δ*agI/II_*1 | ATACCTTCTTCGGAATCTGCT | Construction of S9Δ*agI/II* mutant |
| S9Δ*agI/II_*6 | AAGTAGAATACCAGCTCCAATAAATAGTCCTTCTTTTCTTTTTTATGTAGC | Construction of S9Δ*agI/II* mutant |
| S9Δ*agI/II_*7 | AAAAAGAAAAGAAGGACTATTTATTGGAGCTGGTATTCTACTTGGTA | Construction of S9Δ*agI/II* mutant |
| S9Δ*agI/II_*8 | TTTTT**AAGCTT**GTGAAGTACCTGAACAGGT | Construction of S9Δ*agI/II* mutant |
| pET151_S2*agI/II___*Δ*CWA_*F | CACCGCTGTTGTTGCTTGGGGT | Cloning S2 *agI/II* gene in pET151 plasmid |
| pET151_S2*agI/II___*Δ*LPXTG_*R | CTACTACTAGGCTGAAATTGACACGAGGCCATT | Cloning S2 *agI/II* gene in pET151 plasmid |
| pOri23_S2*agI/II*_EcoRI_F | GGCGC**GAATTC**TCGACGCGGTATAGAATTCCT | Cloning S2 *agI/II* gene in pOri23 plasmid |
| pOri23_S2*agI/II*_PstI_R | GGCGC**CTGCAG**TCAAACCATCTTGGTCTGA | Cloning S2 *agI/II* gene in pOri23 plasmid |
| pOri23_S9*agI/II*_EcoRI_F | GGCGC**GAATTC**TCAACTCGGAAGATAATGCCT | Cloning S9 *agI/II* gene in pOri23 plasmid |
| pOri23_S9*agI/II*_PstI_R | GGCGC**CTGCAG**TCAAACCATCTTGGTCTGA | Cloning S9 *agI/II* gene in pOri23 plasmid |
| Spc^R^_EcoRI_F | GGCGC**GAATTC**GTTCGTGAATACATGTTATA | Amplification of spectinomycin^R^  gene from pSET4s plasmid |
| Spc^R^_EcoRI_R | GGCGC**GAATTC**GTTTTCTAAAATCTGAT | Amplification of spectinomycin^R^  gene from pSET4s plasmid |
