# Supplementary material for: Serotype-specific role of antigen I/II in the initial steps of the pathogenesis of the infection caused by Streptococcus suis
Source: Vet Res. 2017 Jul 14;48:39. doi: 10.1186/s13567-017-0443-4 (PMC5513104; doi:10.1186/s13567-017-0443-4)
Supplement: Supplementary file 3 — Additional file 3. S. suis serotype 2 (S2) and serotype 9 (S9) AgI/II amino acid sequence alignment. Alignment was performed using Vector NTI 11.5. Conserved amino acids appear in light gray and identical amino acids in dark gray. [file 13567_2017_443_MOESM3_ESM.pdf]

```
Section 1
            (1)   1                 10                  20                  30                  40                54
S2 AgI/II   (1)   MTKTCNHHFLVNQEKGEKHVFRKSKKYRTLCSVALGTMVTAVVAWGGTVVHADE
S9 AgI/II   (1)   MTKTCNHHFLVNQEKGEKHVFRKSKKYRTLCSVALGTMVTAVVAWGGTVAHADE
Section 2
            (55)  55        60                  70                  80                  90               108
S2 AgI/II   (55)  VSSSVDTTIQRTENPATNLPEEQPNPVSEQTESLALTGQSNGAIAVTVPHDTVT
S9 AgI/II   (55)  VTSSVDTNIQRTENPATNLPEAQPKPVSEQTEILALTGQSNGAIAVTVPHDTVT
Section 3
            (109) 109                 120                 130                 140                 150        162
S2 AgI/II   (109) QAVEEAKAEGVSTDEDSPMDLGNTTSATETSQQISKAEVDAQNQVEAINEVTET
S9 AgI/II   (109) QAVEEAKAEGVSTVEDSQMDLGNTTSAAETNQQISKAEADAQNQVEAINEVTET
Section 4
            (163) 163           170                 180                 190                 200              216
S2 AgI/II   (163) YKADKATYESNKARIEQENKELSQAYEGVNQTGKETNAWVDTKVNDLKTRYADA
S9 AgI/II   (163) YKADKAAYVDEKARIEQENKELSQAYEGANQTGKETNTWVDTKVKDLKTRYADA
Section 5
            (217) 217                       230                 240                 250                 260  270
S2 AgI/II   (217) DVTVNEQVVSSGNGTAVLDYTNYGKAVETIQSTNEQAVADYLTKKTKADEIVAK
S9 AgI/II   (217) DVTVKEQVVSSGNGTSVLDYTNYGKAVETIQSTNEQAVADYLTKKTKADDIVAK
Section 6
            (271) 271               280                 290                 300                 310        324
S2 AgI/II   (271) NQVIQKENEAGLAKAKADNEAIERRNKAGQAAVDAENRAGQAAVDQANQEKQQL
S9 AgI/II   (271) NQAIQKENEAGLAKAKADNEAIERRNQAGQAAVDAENRAGQAAVDQANQEKQQL
Section 7
            (325) 325       330                 340                 350                 360              378
S2 AgI/II   (325) VSDRAAEIEAITKRNKEKEAAVRKENEAIDAYNAKELECYQRDLAEISKGEEGY
S9 AgI/II   (325) VSDRAAEIEAITKRNQEKEAAARKENEVIDAYNTKEMERYQRDLAEISKGEEGY
Section 8
            (379) 379                 390                 400                 410                 420        432
S2 AgI/II   (379) ISEALAQALNLNNGEPQAQHGANTRNPDQIISTGDALLGGYSRILDSTGFFVYD
S9 AgI/II   (379) ISEALAQALNLNNGEPQAQHGAITRNPNQIISTGDAMLGGYSRILDSTGFFVYD
Section 9
            (433) 433           440                 450                 460                 470              486
S2 AgI/II   (433) SFKTGETLSFNYQNLQNARFDRKKISRVTYDITNLVSPAGTNAVKLVVPNDPTE
S9 AgI/II   (433) SFKTGETLSFNYQNLQNARFDGKKISRVTYDITNLVSPAGTDAVKLVVPNDPTE
Section 10
                  487                       500                 510                 520                 530  540
S2 AgI/II   (487) GFIAYRNDGNGDWRTDRMEFRVVAKYYLEDGSQVTFSKEKPGVFTHSSLNHNDI
S9 AgI/II   (487) GFIAYRNDGNGDWRTDKMEFRVVAKYFLEDGTQVTFSKEKPGVFTHSSLNHNDI
Section 11
                  541               550                 560                 570                 580        594
S2 AgI/II   (541) GLEYVKDSSGKFVPINGSTVQVTNEGLARSLGSNRASDLNLPEEWDTTSSRYAY
S9 AgI/II   (541) GLEYVKDTSGKFVAINGSTVQVTNEGLARSLGFNRASDLNLPEEWDTTSSRYAY
Section 12
                  595       600                 610                 620                 630              648
S2 AgI/II   (595) KGAIVSTVTSGNTYTVTFGQGDMPQNVGLSYWFALNTLPVARTVTPYSPKPHVT
S9 AgI/I    (595) KGAIVSTVTSGNTYTVTFGQGDMPQNVGLSYWFALNTLPVARTLTPYSPKPHVA
Section 13
                  649                 660                 670                 680                 690        702
S2 AgI/II   (649) VDLEPVPEPITVTPDVFTPITFTPEKPVTFTPKPLEEVVQPSLSLTKVTLPVKP
S9 AgI/II   (649) VELEPVPEPITVTPDVFTPKTFTPEKPVTFTPKPLEEVVQPSLTLTKVNLPVKP
Section 14
                  703           710                 720                 730                 740              756
S2 AgI/II   (703) IPKELPTPPQVPTVHYHAYRLTTTPEIMKEVVNSDQANLHEKTVAKDSTVIYPL
S9 AgI/II   (703) IPKELPTPPQVPTVHYHAYRLTTTPEIMKEVVNSDQANLHEKTVAKDSTVIYPL
Section 15
                  757                       770                 780                 790                 800  810
S2 AgI/II   (757) TVDALSPNRAQTTSLIFEDYLPAGYLFDKETTQKENGNYVLGFDETKNFVTLTA
S9 AgI/II   (757) TVDALSPNRAQTTSLIFEDYLPAGYLFDKETTQKENGNYVLSFDATKNFVTLTA
Section 16
```

```
          (811) 811      820       830       840       850          864
S2 AgI/II (811) KENLLQEVNKDLTKVYQLTAPKLYGSVQNDGATYSNSYKLLLNKGTNNAYTVTS
S9 AgI/II (811) KENLLQEVNKDLTKVYQLNAPKLYGSVQNDGATYSNSYKLLLNKGTTNAYTVTS
                                                             Section 17

          (865) 865  870       880       890       900          918
S2 AgI/II (865) NVVTVRTPGDGETTTLITPDKNNENADGVLINDTVVTLGTTNHYRLIWDLDQYK
S9 AgI/II (865) NVVTVRTPGDGETTTLITPDKNNENADSVLINDTVVALGTTNHYRLTWDLDQYK
                                                             Section 18

          (919) 919       930       940       950       960       972
S2 AgI/II (919) GDRSAKETIARGFFFVDDYPEEVLDVVENGTAVTTLEDQKVSGITVKTYASLNE
S9 AgI/II (919) GDRSAKETIARGFFFVDDYPEEVLDVVENGTAVTTLDGQKVSGITVKNYASLNE
                                                             Section 19

                973     980       990       1000      1010        1026
S2 AgI/II (973) APKDLQDKLARAKITPTGAFQVFLPDDNQVFYDQYVQTGTSLALLTKMTVKDSL
S9 AgI/II (973) APKDLQDKLARAKITPTGAFQVFLPDDNQAFYDQYVQTGTSLALLTKMTVKDSL
                                                             Section 20

                 1027         1040      1050      1060      1070      1080
S2 AgI/II (1027) YGPTKTYTNKAYQVDFGNGYETKEVTNTLVSPEPKKQNLNKDKVDINGKPMLVG
S9 AgI/II (1027) YGQTKTYTNKSYQVDFGNGYETKEVTNTLVSPEPKKQNLNKDKVDINGKPMLVG
                                                              Section 21

                 1081     1090      1100      1110      1120        1134
S2 AgI/II (1081) TQNHYTLSWDLDQYRGIKADNSQIAQGFYFVDDYPEEALLPDETAIQFITSDGK
S9 AgI/II (1081) SQNYYTLSWDLDQYRGIKADNSQIAQGFYFVDDCPEEALLPDEAAIQFITSDGK
                                                              Section 22

                 1135 1140      1150      1160      1170           1188
S2 AgI/II (1135) TVSGITVKAYSQLSEAPKTLQAALSKQKIQPQGAFQVFMPEDPQAFFESYVTKG
S9 AgI/II (1135) TVSGITVKAYSQLSEAPKMLQAALSKQKIQPKGAFQVFMPEDPQAFFESYVTKG
                                                              Section 23

                 1189      1200      1210      1220      1230       1242
S2 AgI/II (1189) ENITIVTPMTVLETMLNSGKSYENVAYQVDFGQAYETNTVTNFVPKVTPHKSNT
S9 AgI/II (1189) ENITIVTPMTVLETMLNSGKSYENVAYQVDFGQAYETNTVTNFVPKVTPHKSNT
                                                              Section 24

                 1243   1250      1260      1270      1280          1296
S2 AgI/II (1243) NQEGISIDGKTVLPNTVNYYKIVLDYSQYKDMVVTDDVLAKGFYMVDDYPEEAL
S9 AgI/II (1243) NQEGISIDGKTVLPNTVNYYKIVLDYSQYKDMVVTDDVLAKGFYMVDDYPEEAL
                                                              Section 25

                 1297         1310      1320      1330      1340      1350
S2 AgI/II (1297) TLNPDGIQVLDKDGNRVSGISVSTYASLSEAPKVVQDAMAKRQFTPKGAIQVLS
S9 AgI/II (1297) TLIPDGIQVLDKDGNRVSGISVSTYASLSEAPKVVQDAMAKRQFTPKGAIQVLS
                                                              Section 26

                 1351     1360      1370      1380      1390        1404
S2 AgI/II (1351) SDDPKTFYETYVKTGQTLVVTLPMTVKNELTKTGGQYENTAYQIDFGLAYVTET
S9 AgI/II (1351) SDDPKAYYDTYVKTGQTLVVTLPMTVKNELTKTGGQYENTAYQIDFGLAYVTET
                                                              Section 27

                 1405 1410      1420      1430      1440           1458
S2 AgI/II (1405) VVNNVPKLDPQKDVVIDLSHKDDSLDGKEVALHQTFNYRLVGALIPSNRTTDLF
S9 AgI/II (1405) VVNNVPKLDPQKDVVIDLSHKDESLDGKEVALHQTFNYRLVGAMIPSNRATDLF
                                                              Section 28

                 1459      1470      1480      1490      1500       1512
S2 AgI/II (1459) EYGFEDNYDEKHDEYNGVYRSYLMTDVTLKDGSVLKEGTEVTKYTLQQVDTENG
S9 AgI/II (1459) EYGFEDNYDEKHDEYNGVYRSYLMTDVILKDDSVLKEGTEVTKYTLQQVDTENG
                                                              Section 29

                 1513   1520      1530      1540      1550          1566
S2 AgI/II (1513) LVSISFDKSFLETVSDDSAFQADVYLHMKRIAAGQVENTYLHTVNGYVISSNTV
S9 AgI/II (1513) LVSISFDKSFLETVSDDSAFQADVYLQMKRIAAGQVENTYLHTVNGYVISSNTV
                                                              Section 30

                 1567         1580      1590      1600      1610      1620
S2 AgI/II (1567) VTHTPQPEEPSPNQPTPPQPPIETIEPPVPASILPNTGEQESLLGLIGAGILLG
S9 AgI/II (1567) VTHTPQPEEPSPNQPTPPQPPIESLEPPVPASILPNTGEQESLLGLIGAGILLG
                                                              Section 31

                 1621      1631
S2 AgI/II (1621) TAYGLKKKEEK
S9 AgI/II (1621) TAYGLKKKEEK
```
